# In Silico Mechanistic Profiling to Probe Small Molecule Binding to Sulfotransferases

**DOI:** 10.1371/journal.pone.0073587

**Published:** 2013-09-06

**Authors:** Virginie Y. Martiny, Pablo Carbonell, David Lagorce, Bruno O. Villoutreix, Gautier Moroy, Maria A. Miteva

**Affiliations:** 1 Université Paris Diderot, Sorbonne Paris Cité, Molécules Thérapeutiques In Silico, INSERM UMR-S 973, Paris, France; 2 INSERM, U973, Paris, France; 3 University Evry, iSSB, Évry, France; 4 CNRS, iSSB, Évry, France; Concordia University Wisconsin, United States of America

## Abstract

Drug metabolizing enzymes play a key role in the metabolism, elimination and detoxification of xenobiotics, drugs and endogenous molecules. While their principal role is to detoxify organisms by modifying compounds, such as pollutants or drugs, for a rapid excretion, in some cases they render their substrates more toxic thereby inducing severe side effects and adverse drug reactions, or their inhibition can lead to drug–drug interactions. We focus on sulfotransferases (SULTs), a family of phase II metabolizing enzymes, acting on a large number of drugs and hormones and showing important structural flexibility. Here we report a novel *in silico* structure-based approach to probe ligand binding to SULTs. We explored the flexibility of SULTs by molecular dynamics (MD) simulations in order to identify the most suitable multiple receptor conformations for ligand binding prediction. Then, we employed structure-based docking-scoring approach to predict ligand binding and finally we combined the predicted interaction energies by using a QSAR methodology. The results showed that our protocol successfully prioritizes potent binders for the studied here SULT1 isoforms, and give new insights on specific molecular mechanisms for diverse ligands’ binding related to their binding sites plasticity. Our best QSAR models, introducing predicted protein-ligand interaction energy by using docking, showed accuracy of 67.28%, 78.00% and 75.46%, for the isoforms SULT1A1, SULT1A3 and SULT1E1, respectively. To the best of our knowledge our protocol is the first in silico structure-based approach consisting of a protein-ligand interaction analysis at atomic level that considers both ligand and enzyme flexibility, along with a QSAR approach, to identify small molecules that can interact with II phase dug metabolizing enzymes.

## Introduction

Drug metabolizing enzymes (DMEs) play a key role in the metabolism of endogenous molecules, xenobiotics and drugs introduced into the human body [1–3]. While their principal role is to detoxify organisms by modifying endogenous and exogenous compounds for a rapid excretion, such as pollutants or drugs, in some cases they render their substrates more toxic thereby inducing severe side effects and adverse drug reactions [4–8]. Phase I DMEs catalyze oxidative reactions leading to metabolites that may be either excreted or additionally modified by the phase II DMEs catalyzing conjugation reactions. In some cases, phase II DMEs can directly modify a compound without passing through the phase I DMEs. Overall, most previous investigations have been prioritizing the phase I DMEs, in particular cytochromes P450 (CYPs) [9–12]. Yet, phase II DMEs metabolize a broad range of compounds that can either be beneficial or lead to toxicity, poor drug bioavailability or adverse drug reactions [4–6,13]. Therefore, much efforts are needed to explore their impact on drug efficacy and safety.

**Figure 1:**
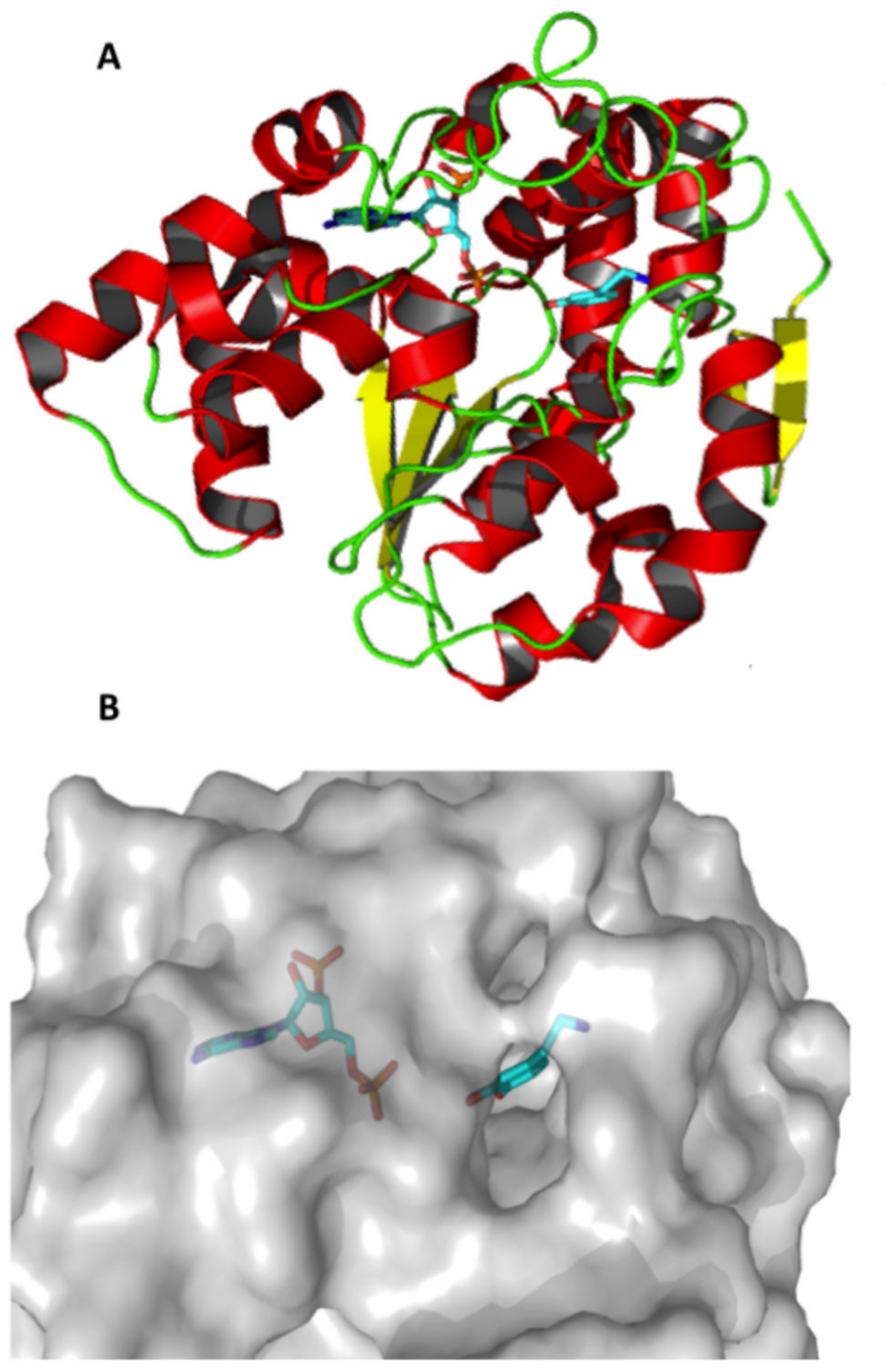
Visualization of the human structure sulfotransferase 1A3 (PDB ID: 2A3R). A: SULT1A3 is represented in cartoon colored according to its secondary structure, red for helices, yellow for strands and green for loops; the co-factor PAP and the co-crystallized ligand L-dopamine are shown in sticks. B: SUTL1A3 is represented in grey surface, with the co-factor PAP and the co-crystallized ligand L-dopamine in sticks.

Here we focus on sulfotransferases (SULTs), a family of enzymes that metabolize a large number of drugs [3]. SULTs [14] (Figure 1) catalyze the sulfoconjugation from the co-factor 3′-Phosphoadenosine 5′-Phosphosulfate (PAPS) to a hydroxyl or amino group of the substrate by executing a nucleophilic attack. At high concentrations some substrates inhibit the enzyme [15] and dead-end complexes with bound inactive cofactor PAP have been identified [15–17]. Sulfoconjugation usually facilitates excretion, but in some particular cases the pharmacological activity of some drugs increases (e.g., the hypotensive prodrug minoxidil becomes fully active after sulfate conjugation). Further, SULTs can convert some chemicals to carcinogens or to activators of promutagens by creating highly reactive sulfate esters that can bind covalently to DNA (e.g. 7,12-dimethylbenz(a) anthracene) [4,6–8]. SULTs that are responsible for the metabolism of small endogenous compounds and xenobiotics are localized in the cytosol [4,8,18]. Four families of human SULTs have been identified by now, SULT1, SULT2, SULT4 and SULT6, and more than 30 X-Ray structures, holo or apo, have been reported in the Protein Data Bank (PDB) [14,19]. Among them, SULT1, metabolizing a wide variety of compounds like phenols, thyroid hormones and drugs (e.g. minoxidil, paracetamol, 17α-ethinylestradiol), is the most expressed one (found in liver, lung, intestine, kidney, thyroid, blood or brain [18]).

Notably, experimental and computational approaches have been proposed to predict Absorption, Distribution, Metabolism, Excretion and Toxicity (ADME-Tox) properties of drugs or the response to environmental toxins [1,20–22]. ADME-Tox predictions [12,22–26] are challenging but extremely important in prioritizing appropriate small molecules not only during the selection of potent candidates in drug discovery projects but also, to some extent, for chemical biology studies. Classical *in silico* ADME-Tox predictions are mostly based on statistical approaches using annotated databases, like Quantitative Structure-Activity Relationships and Quantitative Structure-Property Relationships (QSAR/QSPR) [26–28]. However, the complexity of ADME-Tox molecular mechanisms, for instance specific interactions with DMEs or with other ADME-Tox-related proteins, requires a deep mechanistic understanding [10–12,29] of the ligand-protein interactions at atomic level. Such knowledge should become more accessible for basically all proteins within the next 15 years as structural genomics projects gain full speed [30]. Indeed, in recent years *in silico* approaches exploiting the 3D structure of ADME-Tox related proteins, like docking/scoring or pharmacophore approaches, were successfully developed to complement QSAR models [10–12,29,31–34].

Here we report a novel *in silico* structure-based approach to probe ligand binding to SULTs. We developed a protocol combining docking-scoring methods with QSAR modeling in order to predict SULTs ligand binding. One of the key characteristics of DMEs (CYPs, SULTs) is that they are promiscuous, showing a remarkable plasticity of the active site to adapt its conformation to diverse ligands [12,14,17,35,36]. Therefore, we explored the flexibility of SULTs by molecular dynamics (MD) simulations in order to elucidate the molecular mechanisms involved in ligand binding and to identify the most suitable multiple receptor conformations for ligand binding prediction [29,37–39]. Then, we employed a structure-based docking-scoring approach [34,40,41] to probe ligand binding and finally we combined the predicted interaction energies with a QSAR methodology. To the best of our knowledge our protocol is the first in silico structure-based approach consisting of a protein-ligand interaction analysis at atomic level that considers flexibility, in both the ligands and the enzymes, along with a QSAR approach, to identify small molecules that can interact with II phase DMEs. Our results show that the automated protocol successfully prioritizes potent binders for the three studied here isoforms: SULT1A1, SULT1A3 and SULT1E1. Such approach could be very helpful for drug discovery or chemical biology endeavors, alerting for possible SULTs binding and thus risks of toxicity or poor bioavailability, or for possible strategies for the design of a prodrug.

## Results

### Collecting SULT1 Ligands

We decided to explore three isoforms, SULT1A1, SULT1A3 and SULT1E1, belonging to the largest SULT1 family and for which a number of substrates and inhibitors have been idebntified [14,18,42]. We collected 157, 117 and 80 known binders (substrates or inhibitors) for SULT1A1, SULT1A3 and SULT1E1, respectively (see Materials & Methods section). Several drugs (paracetamol, minoxidil), estrogens and toxic compounds (like bisphenol A used in plastic industry and recently discovered to be toxic) are present in our collection. Chemical structure clustering (see Materials & Methods section for details) resulted in 60, 50 and 33 diverse active compounds for SULT1A1, SULT1A3 and SULT1E1, respectively (examples for each isoform are shown in Table 1 and Figure S1). Putative decoys were taken from the diverse chemical compound libraries ChemBridge™ PremiumSet™ and the Maybridge® HitFinder™. The actives and decoys were merged and filtered using a soft drug-like filter in terms of physicochemical properties (see Materials & Methods section). For each isoform, we obtained two validation datasets, ChemBridge and Maybridge, containing: 46556 and 13148 compounds for SULT1A1; 49546 and 13138 compounds for SULT1A3; 49529 and 13121 compounds for SULT1E1, respectively.

**Table 1:** Examples of diverse active molecules for each SULT1 isoform assigned in different clusters.

Isoform	Compound	Known Function	Type
**SULT1A1**	Diflunisac	Non-Steroidal Anti-Inflammatory Drug (NSAID)	Inhibitor: IC_50_=3.7.10^-6^M
	Bisphenol A	Organic compound raising concerns about its presence in consumer products and foods	Substrate: Km=4.2.10^-6^ M
	Triclosan	Antibacterial and antifungal agent	Inhibitor: IC_50_=2.3.10^-6^ M
**SULT1A3**	Resveratrol	Antioxidant	Substrate: Km=1.3.10^-6^ M
	Curcumin	Curcumin is a curcuminoid of the Indian spice turmeric.	Inhibitor: IC_50_=4.10^-6^ M
	Mefenamic Acid	Non-Steroidal Anti-Inflammatory Drug (NSAID)	Inhibitor: IC_50_=150.10^-9^ M
**SULT1E1**	Daidzein	Daidzein is an isoflavone and acts as antioxidant	Substrate: Km=8.10^-9^ M
	17αethinylestradiol	Ethinyl estradiol is a derivative of estrogen used in oral contraceptive pills	Substrate: Km=3.10^-6^ M
	Trans-Piceatannol	Antioxidant	Inhibitor : Ki=400.10^-9^ M

### Molecular Dynamics Simulations and Multiple Receptor Conformations

**Figure 2:**
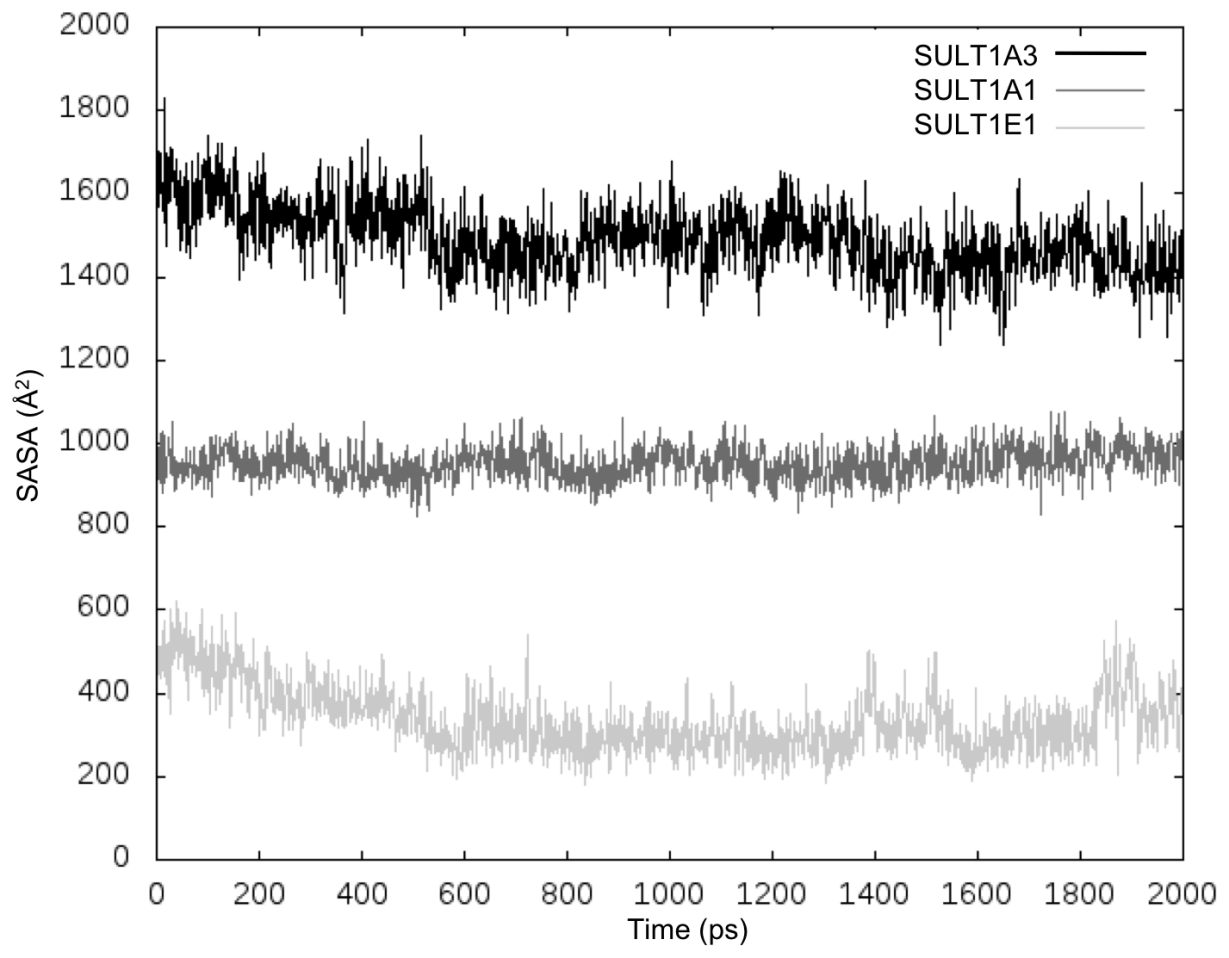
Solvent Accessible Surface Area (SAS) of binding pockets for the three SULT1 isoforms for one MD production.

We ran three MD simulations for each of the isoforms, SULT1A1, SULT1A3 and SULT1E1, with bound cofactor PAP and without bound ligands. Previously, it has been demonstrated that PAPS and PAP have equivalent stabilizing effects on these SULT isoforms [17,43]. Thus, we used PAP (substituting PAPS) in order to generate protein conformations capable to accommodate substrates and inhibitors. All trajectories showed stable potential energies from 0.5 to 2.0 ns of the production range (Figure S2, S3 and S4 given in the supporting information). Our analysis focuses mainly on the plasticity of the binding sites observed during the MD simulations. The list of residues of the binding sites is given in the supporting information (Text S1). The Solvent Accessible Surface Area (SASA) values of the binding sites along the MD production are shown in Figure 2. Among the three isoforms, SULT1A3 displays the highest values of SASA, i.e. the most open binding site, while SULT1E1 displays the lowest values, i.e. the most closed pocket. These results suggest a different dynamic behavior of the binding pockets for the three isoforms.

**Figure 3:**
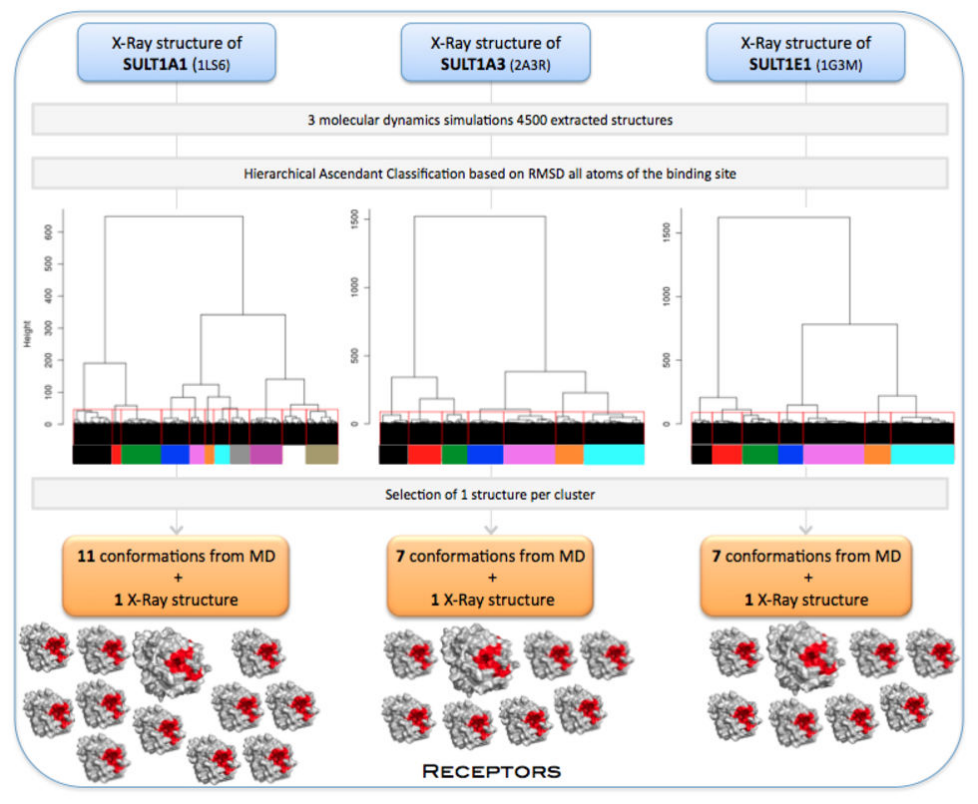
Multiple receptor conformations generation.

For each isoform, we extracted 4500 structures from the three MD productions, from 0.5 to 2.0 ns. In order to select multiple receptor conformations with diverse binding site conformations, we employed Hierarchical Ascendant Classification (HAC) based on the matrix of Root Mean Square Deviation (RMSD) for all atoms of the binding site and of the cofactor. We imposed a RMSD difference of at least 1.3 Å, resulting in 11 conformations for SULT1A1, 7 for SULT1A3 and 7 for SULT1E1 (Figure 3).

### Binding pockets characterization

**Figure 4:**
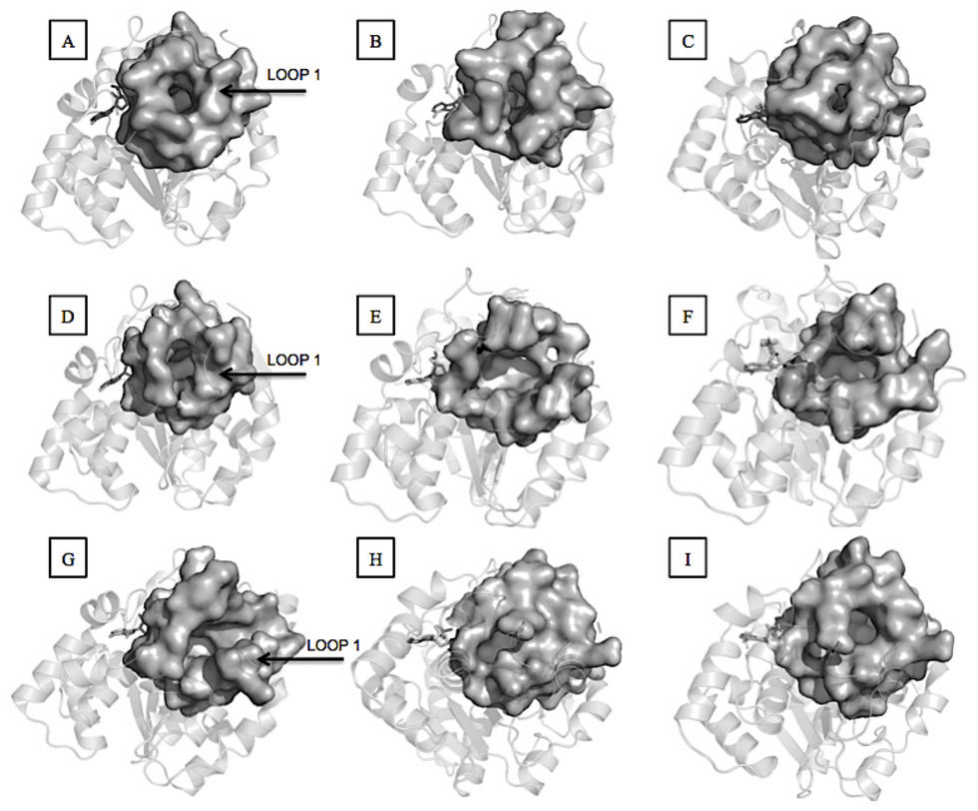
**Binding sites of different SULT1 structures.** A: SULT1A1 X-Ray structure. B: SULT1A1 cluster 4 MD structure. C: SULT1A1 cluster 10 MD structure. D: SULT1A3 X-Ray structure. E: SULT1A3 cluster 3 MD structure. F: SULT1A3 cluster 7 MD structure. G: SULT1E1 X-Ray structure. H: SULT1E1 cluster 3 MD structure. I: SULT1E1 cluster 4 MD structure.

We performed structural analysis of the binding sites of the MD generated conformations and of the X-ray structures (Table 2). The volumes of the binding sites of different SULT1A1 conformations vary from 707.6 to 949.5 Å^3^. We found pockets with volumes quite similar to that of the X-ray structure (for instance clusters 2, 3, 4, 7) and pockets with volumes larger than that of the X-ray structure. Differences were also observed for SASA. For example, the SASA of the pocket of the cluster 4 conformation is similar to that of the X-ray structure (Figure 4A and 4B). In contrast, the cluster 10 conformation (Figure 4C) reveals a closed pocket in terms of SASA. These results indicate that loop 1 may open as a gate (Figure 4A, 4B, and 4C), as previously suggested in [14,44]. The different clusters centroids show various volumes and SASA, suggesting that the binding site of SULT1A1 may easily adapt its conformation to ligands of different sizes and shapes.

**Table 2:** Structural characteristics of the binding sites for different isoforms and multiple receptor conformations.

Isoform	Structure	Pocket Volume (Å^3^)	Pocket Solvent Accessible Surface Area (Å^2^)
**SULT1A1**	**X-Ray**	**735.7**	**963**
	Cluster 1	900.7	940
	Cluster 2	787.9	919
	Cluster 3	707.6	914
	Cluster 4	743.2	925
	Cluster 5	895.5	1009
	Cluster 6	882.3	900
	Cluster 7	781.1	837
	Cluster 8	804.5	894
	Cluster 9	949.5	914
	Cluster 10	894.0	870
	Cluster 11	912.5	859
**SULT1A3**	**X-Ray**	**875.8**	**770**
	Cluster 1	2470.7	1426
	Cluster 2	2749.1	1522
	Cluster 3	2043.4	1425
	Cluster 4	2949.1	1518
	Cluster 5	2588.7	1568
	Cluster 6	2440.0	1455
	Cluster 7	1958.8	1338
**SULT1E1**	**X-Ray**	**1118.8**	**379**
	Cluster 1	1385.7	356
	Cluster 2	873.8	274
	Cluster 3	952.7	290
	Cluster 4	1337.5	399
	Cluster 5	1774.8	516
	Cluster 6	2023.6	620
	Cluster 7	2123.8	637

Results for SULT1A3 are unexpected. As seen from Table 2, all obtained clusters centroids have huge binding sites with volume three times larger than that of the X-ray structure while SASA can be twice higher than in the X-Ray structure. The structural comparison (Figure 4D, 4E, 4F and Figure 2) shows that the binding site remains open with large volume and SASA during the entire MD trajectories. It is likely that the bound L-dopamine in the X-ray structure maintains the pocket conformation more closed than in the modeled MD apo structures, e.g. an induced fit is present in the complex, as similar phenomena have already been observed in other protein-ligand complex structures [45]. Indeed, the amino group of L-dopamine is located between the two carboxylic groups of Glu146 and Asp86, thus involved in two salt bridges. During the MD simulations without bound ligand, the repulsing interaction between the two carboxylic groups led to the opening of loop 1 (amino acid residues 85-90). Interestingly, the superposition of the two available X-ray structures for SULT1A3, one bound with L-dopamine (PDB ID 2A3R) [46] and one apo (PDB ID 1CJM) [47], shows a displacement of loop 1 of 4.9 Å. Thus, the substrate-binding pocket is largely open in the X-ray apo structure likewise in the modeled MD apo structures. The conformational change of loop 1 of SULT1A3 accompanying the ligand binding [14,44] is strongly supported by the holo and apo X-ray structures and the MD apo conformations.

Next, the MD structures of SULT1E1 show diverse pocket conformations, larger or smaller than the X-ray one (Figure 4G, 4H, 4I and Figure 2). SULT1A1 and SULT1E1 have some similarities regarding their binding sites plasticity. For the two isoforms, the binding pockets of the representative MD structures are either larger or smaller than those of the X-Ray structures. Thus, these isoforms can accommodate their binding sites according to the ligands size.

Some differences of the binding sites for different SULT1 isoforms observed here could explain some of the substrate specificities known for the three isoforms. Overall, the volumes of different binding site conformations of SULT1E1 are larger than those of SULT1A1. The larger pocket of SULT1E1 may facilitate its favorable interactions with large ligands. Indeed, steroids, which are large and quite rigid molecules, are specific substrates for SULT1E1. Further, all MD centroid structures for SULT1A3 have significantly larger binding pockets than the holo X-ray structure. As such, opening of the binding site may facilitate interactions with more bulky ligands than the co-crystallized dopamine. In fact, large ligands also interact with SULT1A3, for instance α-zearalenol, dobutamine or SKF38393. However, experimental SULT1A3 structures with bound large ligands would be very helpful to clarify the ligand-binding mechanism for this isoform.

### Identifying the Best Multiple Receptor Conformations by Virtual Screening

**Figure 5:**
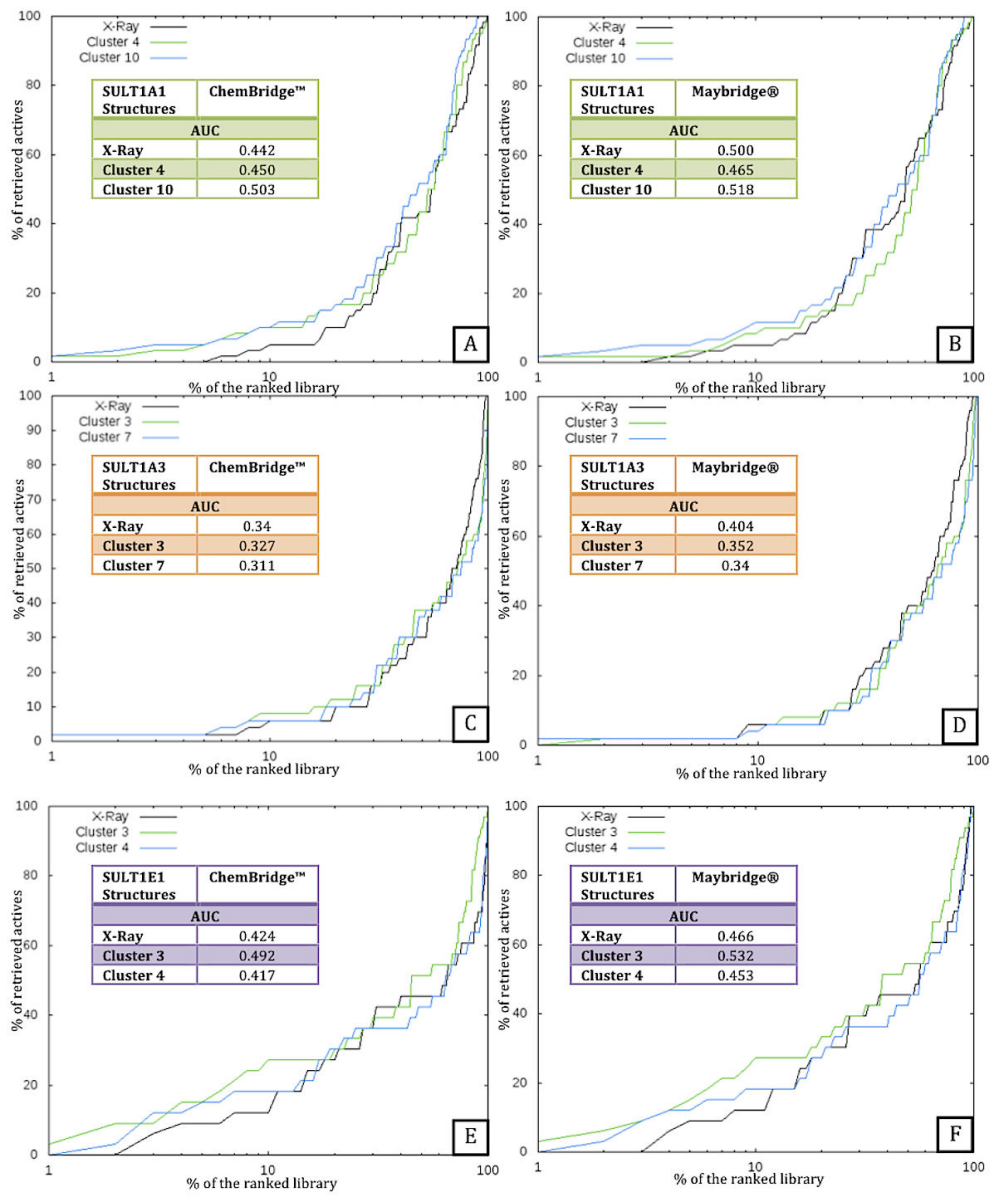
Enrichment graphs obtained on the X-ray and two best performing MD structures for each isoform. The X-Ray structure is in black and the MD structures in blue and green. 100% refers to all screened compounds including all actives and decoys. The Receiver Operating Characteristic Curve Areas (AUC) are also given. A: SULT1A1 with ChemBridge™ as decoys. B: SULT1A1 with Maybridge® as decoys. C: SULT1A3 with ChemBridge™ as decoys. D: SULT1A3 with Maybridge® as decoys. E: SULT1E1 with ChemBridge™ as decoys. F: SULT1E1 with Maybridge® as decoys.

Virtual Screening (VS) experiments were performed using docking-scoring approach in order to identify the protein conformations, which can better discriminate known binders from putative decoys. We ran 56 VS for all isoforms. A total of 24 VS were carried out for SULT1A1 on the 11 representative MD conformations and the X-ray structure with two compounds datasets, ChemBridge and Maybridge. Similarly, we performed 16 VS for SULT1A3 and 16 VS for SULT1E1. The best results obtained for the MD centroid conformations and the X-ray structures for each isoform are shown by enrichment graphs in Figure 5. Protein-ligand interaction energies for active centroids as predicted by docking (averaged for the X-ray and the two best performing MD structures) and compared to the experimental affinities are given for illustration in Table S1 (see the Supporting Information). As it is widely accepted, docking approaches cannot achieve an exact prediction of the binding affinities, rather they help to prioritize active molecules among a large number of compounds.

Two MD structures for SULT1A1 achieve better enrichment results than the X-ray structure on the ChemBridge™ dataset (Figure 5A). Indeed, the structures of clusters 4 and 10 show Area Under Curve (AUC) for the Receiver Operating Characteristic (ROC) curve (not shown) larger than the X-ray structure. The early enrichments are also better for these two MD structures than for the X-ray one. Similar results are obtained on the Maybridge® dataset (Figure 5B). The best performing is the cluster 10 structure adopting slightly larger binding pocket than the holo X-ray structure allowing thus better prediction for the larger actives. In the case of SULT1A3 (Figure 5C and 5D) the VS experiments did not suggest a MD structure performing better than the holo X-ray one, yet early enrichments are slightly better for two MD structures than for the X-ray one in the case of the ChemBridge™ dataset. The MD structures and the holo X-ray for SULT1A3 show similar AUC, lower than those for SULT1A1. The best MD structures for SULT1A3, clusters 3 and 7, have binding sites twice larger than the holo X-ray one, thus, not allowing to correctly rank the small actives and prioritizing large ligands, actives or decoys. Our analysis showed a balanced representation of small and large actives for SULT1A3 in our datasets (see Dataset 1. sdf for SULT1A1, Dataset 2. sdf for SULT1A3 and Dataset 3. sdf for SULT1E1 given in the supporting information). Next, very good results have been obtained for SULT1E1 (Figure 5E and 5F). The two best MD structures demonstrate much better early enrichment and larger AUC in comparison to the X-ray structure. The best performing is the cluster 3 structure showing slightly smaller volume of the binding site than the X-ray one.

**Table 3:** Druggability scores for different isoforms and conformations.

Isoform	Structure	Druggability
**SULT1A1**	**X-Ray**	**0.91**
	Cluster 1	0.92
	Cluster 2	0.77
	Cluster 3	0.62
	Cluster 4	0.88
	Cluster 5	0.81
	Cluster 6	0.83
	Cluster 7	0.63
	Cluster 8	0.82
	Cluster 9	0.94
	Cluster 10	0.97
	Cluster 11	0.85
**SULT1A3**	**X-Ray**	**0.92**
	Cluster 1	0.41
	Cluster 2	0.32
	Cluster 3	0.36
	Cluster 4	0.43
	Cluster 5	0.62
	Cluster 6	0.81
	Cluster 7	0.79
**SULT1E1**	**X-Ray**	**0.91**
	Cluster 1	0.78
	Cluster 2	0.97
	Cluster 3	0.96
	Cluster 4	0.94
	Cluster 5	0.46
	Cluster 6	0.41
	Cluster 7	0.23

Overall considering the flexibility of the binding sites via multiple receptor conformations improved the enrichments. We achieved better discrimination for binders of SULT1A1 and SULT1E1 than for SULT1A3. The discrimination performance for SULT1A3 is lower because during the MD simulations this isoform adopted large and open binding site conformations due to the two closely placed Glu146 and Asp86 in the initial structure. On the other hand, the holo X-ray binding pocket is extremely closed, therefore not allowing correct positioning and ranking of large active molecules. In both cases, the enrichment is worsened in comparison with the other isoforms.

We also analyzed the druggability of the MD and X-ray structures. We calculated the druggability score using DogSiteScorer [48]. DogSite calculates several pockets descriptors and employs support vector machine method to return a score of druggability between 0 and 1 (0 – non-druggable, 1 - druggable). A strong druggability score (>0.9) was attributed to the X-ray structures for the three isoforms (see Table 3). Interestingly, our multiple receptor protocol successfully provides 3 MD conformations with a higher druggability score than the X-ray structures for SULT1A1 and for SULT1E1. Moreover, the conformations best discriminating the actives for SULT1A1 and SULT1E1 show druggability scores of 0.97 and 0.96, respectively. Further, lower druggability scores were obtained for the MD conformations of SULT1A3 as compared to the X-ray structure. Despite of the very high druggability score of the X-ray SULT1A3, the obtained enrichment is not very high. Thus, the druggability score is a useful indicator but is not sufficient for a final selection of the best multiple receptor conformations, a validation with known binders can be critical.

**Figure 6:**
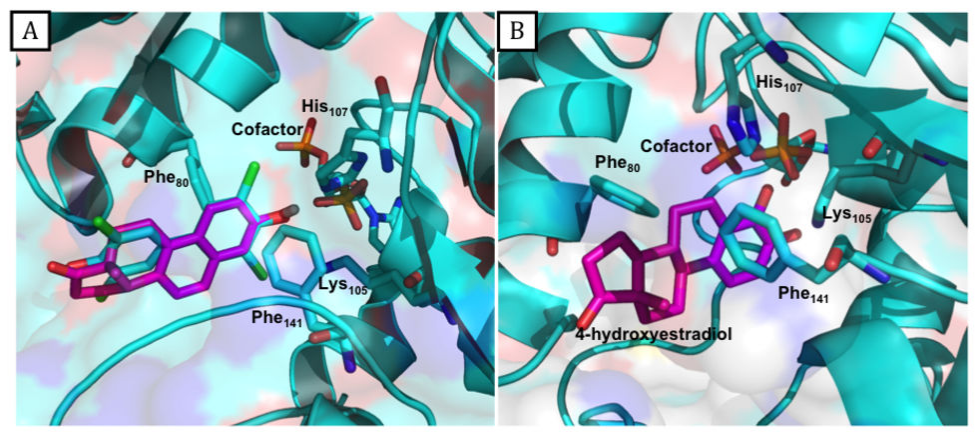
Best scored poses for SULTE1 actives. A: X-Ray structure of SULT1E1 with the co-crystallized ligand (3,5,3',5'-tetrachloro-biphenyl-4,4'-diol) in cyan and the best scored pose of the docked equilenin in magenta. B: Best scored pose of the best ranked compound 4-hydroxyestradiol in magenta docked into the MD Cluster 3 structure.

To exemplify the predicted protein-ligand interactions, we focused on the first retrieved actives for SULT1E1, known to metabolize estrogen hormones. The first actives ranked by the employed docking-scoring were the estrogen derivatives equilenin and 4-hydroestradiol for the X-ray structure and for the cluster 3 MD structure, respectively (Figure 6). We compared the best scored pose of equilenin to the bioactive conformation of the co-crystallized ligand 3,5,3',5'-tetrachloro-biphenyl-4,4'-diol. Figure 6A shows that equilenin and 3,5,3',5'-tetrachloro-biphenyl-4,4'-diol are well superimposed and that the hydroxyl group is correctly oriented in the docked pose. Indeed, the ligand hydroxyl group points toward the co-factor close to the phenylalanine gates Phe80 and Phe141 and forms a hydrogen bond with H107, which is believed to act as a catalytic base deprotonating the hydroxyl group during the sulfate transfer [49]. Docking of the best-ranked 4-hydroxyestradiol into the MD cluster 3 structure was also successful (Figure 6B), the ligand hydroxyl group, supposed to be sulfated, points toward the co-factor, close to the phenylalanine gates Phe80 and Phe141.

### New QSAR models for SULT1

We considered further the molecular structures of the SULT1 active compounds in order to train QSAR classification models. For each isoform, we built two models: a model based solely on topological information of compound structures using extended connectivity fingerprint descriptors (ECFPs) [50] and another model that combined such information with the binding energies computed on the MD protein conformations that best retrieved the active compounds. Such information was then employed to train classifiers using three machine learning methods: support vector machine-based (SVM), random forest and naïve Bayes. The models obtained with the support vector machine gave slightly better performances (see Table S2). Table 4 shows the performance of the SVM classifier with and without included predicted binding energy values. For SULT1E1, we obtained an accuracy of 73.94% (the percentage of correctly predicted active compounds) in a leave-one-out (LOO) cross-validation of the training set, which was increased to 75.46% when introducing the energy values as an additional input feature of the training set. In the case of SULT1A3, the accuracy was raised from 73.80% to 78.00% when using the predicted interaction energies. The QSAR models for SULT1A1 showed lower performance; the obtained accuracy in the leave-one-out cross validation of 60.85% using ECFPs was increased to 67.28% when binding energy information was added. The lower accuracy of SULT1A1 models might be due the higher chemical diversity of the active compounds for this isoform. In all cases, the validation test performed on the external set was successful, with more than 80% of the active compounds correctly classified when using the models with the best performance. The high performance on the external sets might be due to the fact that the external sets contain compounds similar to those of the training set within a Tanimoto similarity of 0.6 (FCFP_4). Therefore our models have been here validated within a domain of applicability given by the clustering threshold.

**Table 4:** Performance of the SVM QSAR model for each isoform.

Isoform	Accuracy of QSAR model without binding energy information (LOO cross validation)	Accuracy of QSAR model including binding energy information (LOO cross validation)	Accuracy (external set)	Size of the training set	Size of the external set
SULT1E1	73.94%	75.46%	95.24%	120	86
SULT1A3	73.80%	78.00%	89.53%	100	66
SULT1A1	60.85%	67.28%	85.85%	66	42

## Discussion

Phase II DMEs encounter drugs or other xenobiotics, in general, modifying them into more hydrophilic metabolites to be easily eliminated from the human body. However, highly reactive, mutagenic or carcinogenic metabolites can also be produced [4–8]. Phase II DMEs have up to now attracted much less attention than cytochromes P450 [3,29,51] despite of their critical roles in detoxification. For instance, UGTs and SULTs, two major DMEs, are involved in the metabolism of many clinically used drugs [3]. Inhibiting SULTs can alter their natural functions that can cause drug–drug interactions [16,29,52]. All together these data demonstrate the importance of exploring phase II DMEs. As several X-ray structures for these enzymes are available, such investigation can be carried out at the atomic level. In the present study, we aim at developing a method for prediction of interactions of these enzymes with putative ligands, drugs or xenobiotics like pollutants.

To this end, we developed a novel approach to predict small molecules binding on three main SULT1 isoforms by combination of a docking-scoring approach, which takes into consideration the flexibility of the protein binding site, and QSAR modeling. Indeed, the SULT1 active sites accommodate very diverse ligands in terms of size and chemistry, thus, it is of crucial importance to introduce possible conformational changes for ligand binding prediction. Although many experimental 3D structures of SULTs have been resolved, only a few 3D *in silico* studies have been reported to date [42–44]. These studies were not fully focused on the exploration of SULT to integrate the protein flexibility for predicting ligand-target interactions for a large number of compounds. Conformational changes observed in the binding sites of SULT1 family suggest that gating of loop 1 can be involved in the ligand binding [14,17,44]. Overall, our MD simulations support similar movements. However, more complex ligand-binding mechanisms might be possible for SULT1A3. It was suggested that substrates may not be completely uncoupled from binding of the cofactor [17].

The better enrichments that we obtained using the MD best structures compared to the rigid X-ray structures confirm that it is indeed critical to take into account the flexibility of the binding sites for SULT1 in order to better discriminate binders from non-binders. The conformational changes of the binding sites of SULT1, e.g. loop 1 gating, facilitate the binding of diverse ligands, which is of major importance for the SULTs role, i.e. the detoxification. We achieved very good predictive results using docking-scoring into the best MD conformations for SULT1A1 and SULT1E1. Recently, Stjernschantz and co-workers [43] screened experimentally 34 potential endocrine-disrupting compounds on the murine and human SULT1E1 to identify selective inhibitors of the human enzyme. They then docked the identified active compounds using the software GOLD [53] and performed subsequent MD simulations of the docked complexes. This process helped to explain in part the selectivity for some of the inhibitors of human SULT1E1. Using our VS docking experiments we retrieved the most potent inhibitors identified in [53] (estrogen derivatives) as the top ranked compounds, confirming the very good prediction performance of our approach for SULT1E1.

Regarding the SULTs substrate diversity and specificities, several SULTs families have been studied by hierarchical clustering of the chemical structures. Two studies, exploring the similarity for local sequences and structural features of the binding site and compound activity profiles [17,54], suggested that SULT1A1 and SULT1A3 could display different substrate specificities due to local sequence and structural differences of the binding sites. As mentioned above, two negatively charged groups are present in the binding site of SULT1A3, Asp86 and Glu146, while two hydrophobic Ala residues are located in the same positions in SULT1A1. Yet, the authors highlighted the difficulty of predicting small-molecule binding patterns for SULT family only from sequence or structural analysis [54]. It is interesting to note that we obtained better QSAR prediction for SULT1A3 than for SULT1A1 suggesting that SULT1A1 is more promiscuous than SULT1A3. These results are consistent with our MD simulations for SULT1A1 suggesting that its binding site is very flexible as adopting open or closed conformations along the MD trajectories that would facilitate the accommodation of diverse ligands. Indeed, SULT1A1 interacts with a large diversity of compounds, including simple phenols, polycyclic aromatic compounds, estrogens. Further, Schapira et al. [42] performed VS experiments using docking of 50,000 compounds (including drugs, clinical candidates and endogenous molecules) into SULT1A3 and SULT1E1 in order to explore substrate selectivity profile. They distinguished correctly the preferential substrate classes for the two isoforms, i.e. catecholamines (e.g. L-dopamine) for SULT1A3 and steroids for SULT1E1. All docking results were carried out into rigid X-ray structures extracted from X-ray structures co-crystallized with the same preferred class of compounds, however, considering the receptor flexibility would be crucial to predict binders structurally different from the co-crystallized ligands.

In order to identify structural factors of substrates controlling the sulfonation several QSAR models have been developed for SULT [55–57]. Taskinen et al. [58] built a QSAR model for SULT1A3 based on 53 catechol compounds using Partial Least Square (PLS). They found that the lipophilicity was positively correlated with sulfonation rate, but specific effects of polar functional groups were more important than the general lipophilicity. In particular, the presence of a positively charged amino group favored sulfonation, whereas the presence of a carboxylate anion strongly decreased reactivity. Finally, the authors obtained a QSAR model with Q^2^ value of 0.72. The same group developed 3D QSAR models performing comparative molecular field analysis (CoMFA) on 95 diverse compounds, obtaining in this case a Q^2^ value of 0.624 for the best model [55]. The two negatively charged residues Glu146 and Asp86 of the binding site explain the preference for a positive charge in the substrate. Such properties of the binding site are explicitly taken into our QSAR model via the predicted protein-ligand binding energy. We achieved 89.53% prediction accuracy for the external set of 66 active compounds for SULT1A3. In fact, the predicted binding energies improved the QSAR models accuracy for the three isoforms highlighting thus the complementarity of the structure-based and QSAR approaches.

We should note that SULTs have not been extensively experimentally screened and we did not find real decoys in publicly available chemistry databases and in several commercial collections (see the Methods section). Therefore, developing *in silico* models, structure-based or QSAR, to predict ligand binding for SULTs is challenging, it might be possible that some of the used putative decoys are active for some SULT isoforms. Yet, our results demonstrate that the developed approach can be successfully employed to predict ligand binding to the three SULT1s isoforms studied here. Resulting in accurate QSAR models based on structure-based methodology, our approach constitutes a major advance in the *in silico* prediction of ADME-Tox properties of small molecules related to interactions with phase II DMEs.

## Material and Methods

### Compound datasets preparation

**Figure 7:**
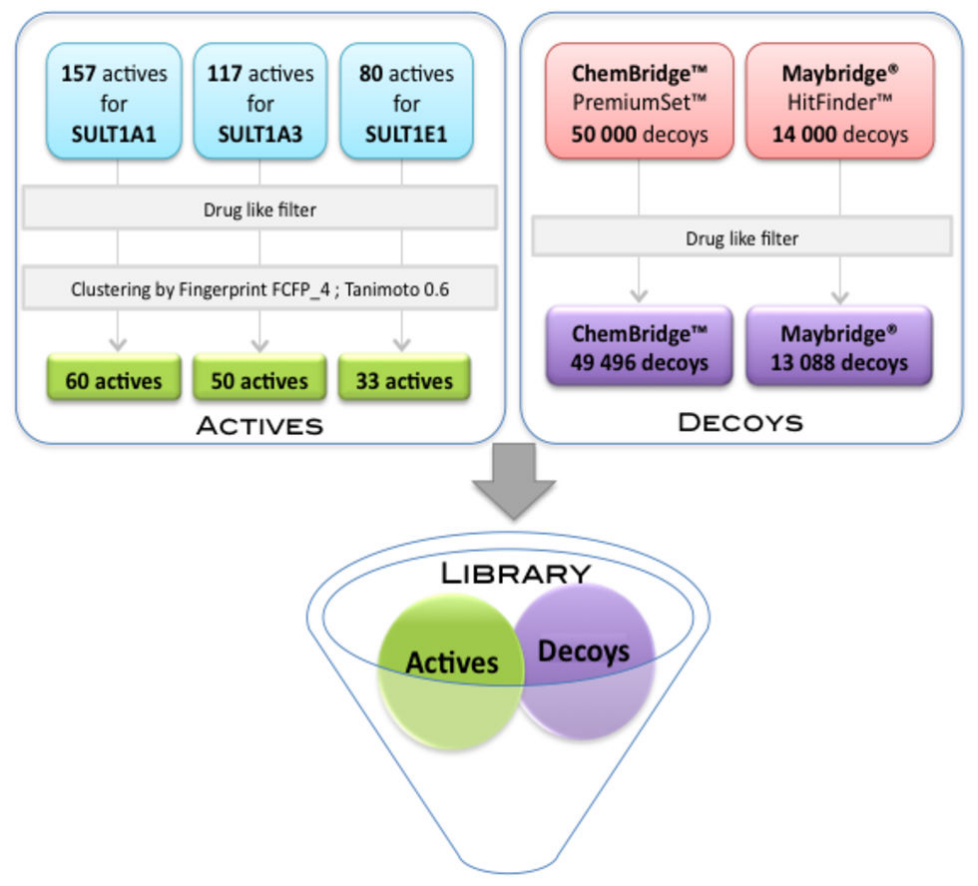
Compound datasets preparation.

Small molecules known to bind to SULT1A1, SULT1A3 and SULT1E1, substrates or inhibitors, were collected (Figure 7) from the databases BRENDA [59], Aureus Sciences (http://www.aureus-sciences.com/), TOXNET (http://toxnet.nlm.nih.gov/), PubChem [60] and literature [6,17,18]. We did not found experiment data for inactive molecules, thus we took putative decoy molecules those from two diverse chemical compound collections, ChemBridge™ PremiumSet™ (http://www.chembridge.com/) and the Maybridge® HitFinder™ (http://www.maybridge.com/) composed of 50000 and 14000 compounds, respectively. In order to select only drug-like molecules, all datasets of actives and putative decoys were filtered using the FAF-Drugs 2 server [61] using “soft” drug-like physicochemical properties (see Text S1 in supporting information) while the search utility to remove toxic/reactive groups was turned off. After filtering, 49496 and 13088 decoys remained from the ChemBridge™ and Maybridge® datasets, respectively. In order to select chemically diverse molecules for validation of our approach and to avoid possible over-representation of a chemical series, we performed several initial tests to cluster the actives using the fingerprints ECFP_4, ECFP_6, FCFP_4 and FCFP_4 as implemented in Pipeline Pilot v.7.5 (SciTegic, Inc/Accelrys). Finally, the filtered drug-like actives were clustered using FCFP_4 with a Tanimoto similarity criterion of 0.6. We then created two compound datasets for each SULT1 isoform taking the active centroids of each cluster for the corresponding isoform, and the decoys from the Chembridge™ et and the Maybridge® filtered datasets, respectively.

### Protein structures preparation

For the three most important SULT1 isoforms, SULT1A1, SULT1A3 and SULT1E1, we selected X-ray structures with co-crystallized ligands and as complete as possible. For SULT1A1, 5 holo PDB structures are available, all sharing very similar conformations (all atom RMSD between the 5 structures vary between 0.170 and 0.239 Å). The visual analysis of these 5 structures did not show striking differences in the binding sites. For SULT1A3, only 1 holo structure is available. The second one is an apo-form with many missing residues around the cofactor binging site. For SULT1E1, only 1 holo structure is available. Thus, we took the X-ray structures co-crystallized with the ligands: p-nitrophenol (PDB ID: 1LS6 [6]), L-dopamine (PDB ID: 2A3R [46]) and 3,5,3',5'-tetrachloro-biphenyl-4,4’-diol (PDB ID: 1G3M [62]), for the isoforms SULT1A1, SULT1A3 and SULT1E1, respectively. All ligands and water molecules were removed. We kept PAP as cofactor for all MD and docking simulations. The pK_a_ values of titratable groups were calculated with the Finite Difference Poisson Boltzman approach [63] using the web server tool Protein Continuum Electrostatics (PCE) [64] with the default parameters (dielectric value of 4 and 80 for solute and solvent, respectively). No abnormal titration behaviors were obtained and His protonation was assigned according to the computed pKas.

### Molecular dynamics simulations

For each SULT1 isoform we performed three molecular dynamic (MD) simulations using CHARMM c35b1 version [65]. We used the all atoms PARAM27 force field [66,67]. All simulations were performed using monomer structures because previous MD studies for SULT1A1 and SULT1E1 suggested identical behavior for monomers and dimers [43,44]. For each SULT1 structure we kept the cofactor PAP. Topology and parameters of PAP were assigned by using the web server SwissParam [68]. The solvation was taken into account by the Generalized Born implicit solvent function FACTS [69]. Non-bonded interactions were truncated in a cut-off distance of 12 Å with a shift function for electrostatics and switch function for the van der Waals interactions. The protein structures were initially minimized using 500 steps of steepest descent algorithm followed by 500 steps of conjugate gradient algorithm. Distances between heavy atoms and hydrogen atoms were constrained by SHAKE algorithm [70] allowing a time step of 2 fs. The system was heated during 100 ps to reach 300 K and then equilibrated during 200 ps with a temperature window of 300±10 K. The production time was 2 ns for each MD simulation run. We have 3 trajectories per isoform at different initial velocities.

### Multiple receptor conformations selection

For each isoform we extracted 4500 structures from the three merged MD trajectories. SASA of the binding sites along the MD trajectories was calculated using CHARMM program and the volume was calculated using CASTp web server [71]. For each isoform the RMSD between the 4500 structures were calculated for all atoms of the binding site and of the cofactor (see Text S1 in the supporting information). We clustered different conformations of the binding sites by applying HAC on the obtained RMSD matrix using the aggregative method ward as implemented in R software [72] and a RMSD distance of at least 1.3Å. We took the centroid structure of each cluster in order to define a representative set of protein conformations for subsequent validation by virtual screening experiments.

### Virtual screening experiments

First we performed preliminary docking experiments with DOCK6.0 [73], AutoDock [74] and Vina 1.1.1 [75], to dock several small and large ligands, dopamine, 4-nitrophenol, pentachlorophenol, estradiol and 3,5,3',5'-tetrachloro-biphenyl-4,4'-diol, into the three isoforms. We obtained the best docking results using Vina with a RMSD of the bioactive conformations within 1Å. Thus, for the subsequent prediction of the SULTs binders, we performed VS experiments using Vina. We used grid resolution of 1 Å, number of binding modes of 10 and exhaustiveness of 8. Fifty six VS runs were performed on X-ray structures and protein structures extracted from MD for the two datasets, the ChemBridge™ and the Maybridge®, containing 49496 and 13088 compounds, respectively. For SULT1A1, the grid size was of 24 Å for the three X, Y, Z axes and the center coordinates were set to 22.979, 105.852 and 57.607. For SULT1A3, the grid size was of 26 Å, 24 Å and 30 Å for the three X, Y, Z axes, respectively, and the center coordinates were set to 56.936, 120.268 and -0.116. For SULT1E1, the grid size was of 18 Å, 22 Å and 22 Å for the three X, Y, Z axes, respectively, and the center coordinates were set to -4.619, -16.110 and 33.948.

### QSAR classification model of active compounds

The centroids of the clustered active compounds known to bind SULT1A1, SULT1A3 and SULT1E1 (Figure 7) were selected as positive sets in order to train QSAR classification models for compounds binding SULTs. The resulting size was 60 for 1A1, 50 for 1A3 and 33 for 1E1. For each positive set of active compounds, we built balanced training sets by random sampling of negatives from the list of the 13088 putative decoys of the Maybridge® library. Thus, the resulting training sets contained 120, 100, and 66 molecules for 1A1, 1A3, and 1E1, respectively (Table 4). The process was repeated 10 times for each isoform in order to compute average results from multiple random samples of the negative set. To describe topological features of the compounds we used extended connectivity fingerprints (ECFPs) [50] up to an atom vicinity of 2. To reduce the dimensionality of the resulting input feature matrix, we applied principal component analysis using the statistical package R.

Then we used three machine learning methods: a support vector machine by using the kernlab R package (ksvm function) [76], a random forest-based predictor by using the randomForest R package [77], and a naïve Bayesian predictor by using the caret R package [78]. For each of them we built two QSAR models: for the first one we used only ECFP descriptors, while for the second one the protein-ligand binding energies calculated on the best performing MD receptor conformation were added as an additional descriptor. Performance of each QSAR classification model was assessed by the percentage of correctly classified compounds in comparison to the total number of compounds in the set through leave-one-out cross-validation. In addition, we used an external validation dataset that contained all known active compounds after the FAF-Drugs 2 filtering, where the molecules identical with those of the training set were removed (resulting in 86 compounds for 1A1, 66 compounds for 1A3, 42 compounds for 1E1).

## Supporting Information

Dataset S1 **Active compound centroids for SULT1A1.**
(SDF)

Dataset S2 **Active compound centroids for SULT1A3.**
(SDF)

Dataset S3 **Active compound centroids for SULT1E1.**
(SDF)

Figure S1 **2D structure of diverse active molecules for each SULT1 isoform.**
(TIFF)

Figure S2 **Potential energies (kcal/mol) for SULT1A1 isoform.** **A**: The potential energy of the MD run 1. **B**: The potential energy of the MD run 2. **C**: The potential energy of the MD run 3.
(TIFF)

Figure S3 **Potential energies (kcal/mol) for SULT1A3 isoform.** **A**: The potential energy of the MD run 1. **B**: The potential energy of the MD run 2. **C**: The potential energy of the MD run 3.
(TIFF)

Figure S4 **Potential energies (kcal/mol) for SULT1E1 isoform.** **A**: The potential energy of the MD run 1. **B**: The potential energy of the MD run 2. **C**: The potential energy of the MD run 3.
(TIFF)

Table S1 **Protein-ligand interaction energies for active centroids as predicted by docking, averaged for the X-ray and the two best performing MD structures, and compared to the experimental affinities.**
(DOCX)

Table S2 **Performance of QSAR models built by using support vector machine, random forest, and naïve Bayes machine-learning methods.**
(DOCX)

Text S1 **Drug-like filter parameters and binding sites’ residues.**
(DOCX)
