# Supplementary figures and images for: In Silico Mechanistic Profiling to Probe Small Molecule Binding to Sulfotransferases
Source: PLoS One. 2013 Sep 6;8(9):e73587. doi: 10.1371/journal.pone.0073587 (PMC3765257; doi:10.1371/journal.pone.0073587)

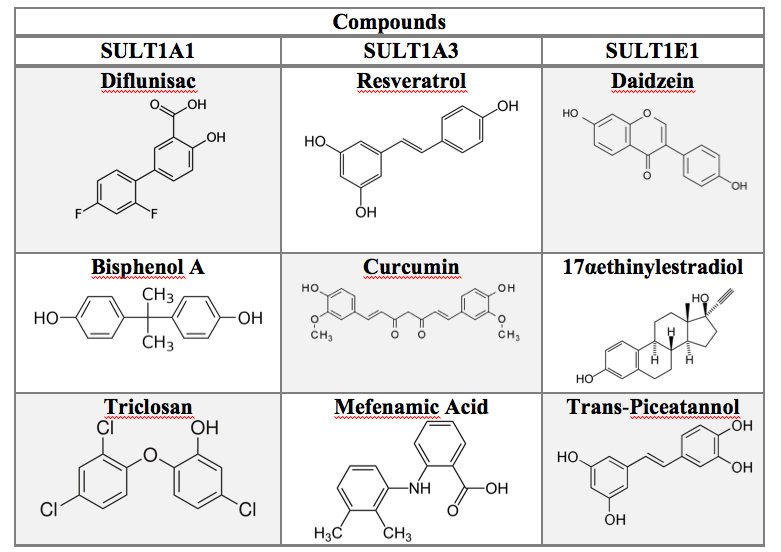

Supplement: Figure S1 — 2D structure of diverse active molecules for each SULT1 isoform. (TIFF) [file pone.0073587.s004.tiff]

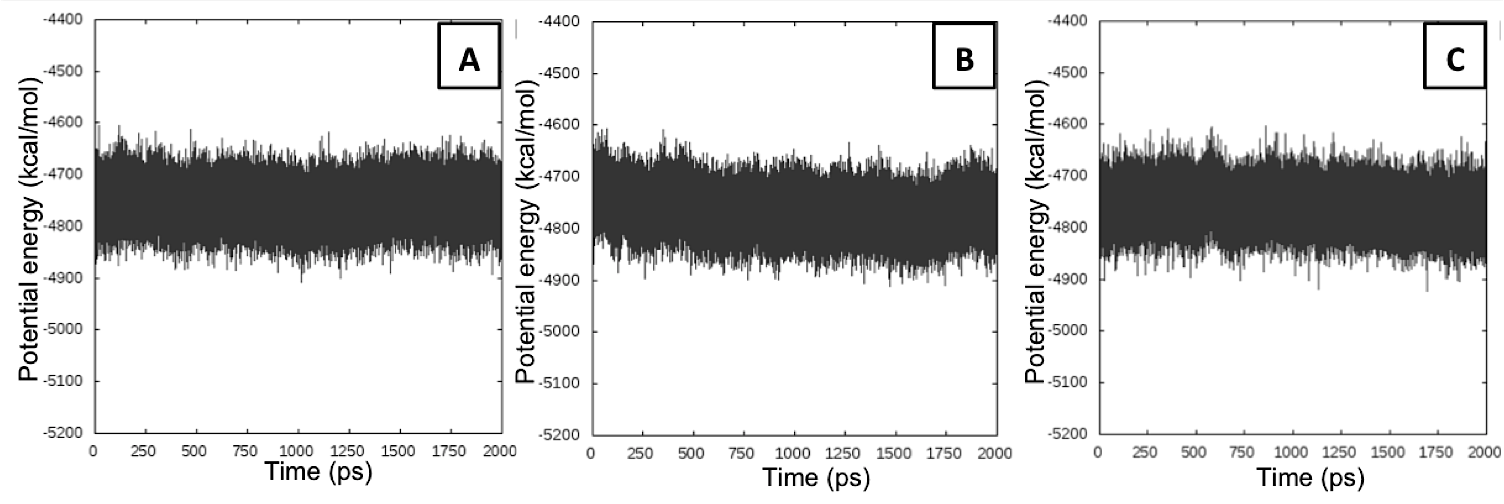

Supplement: Figure S2 — Potential energies (kcal/mol) for SULT1A1 isoform. A: The potential energy of the MD run 1. B: The potential energy of the MD run 2. C: The potential energy of the MD run 3. (TIFF) [file pone.0073587.s005.tiff]

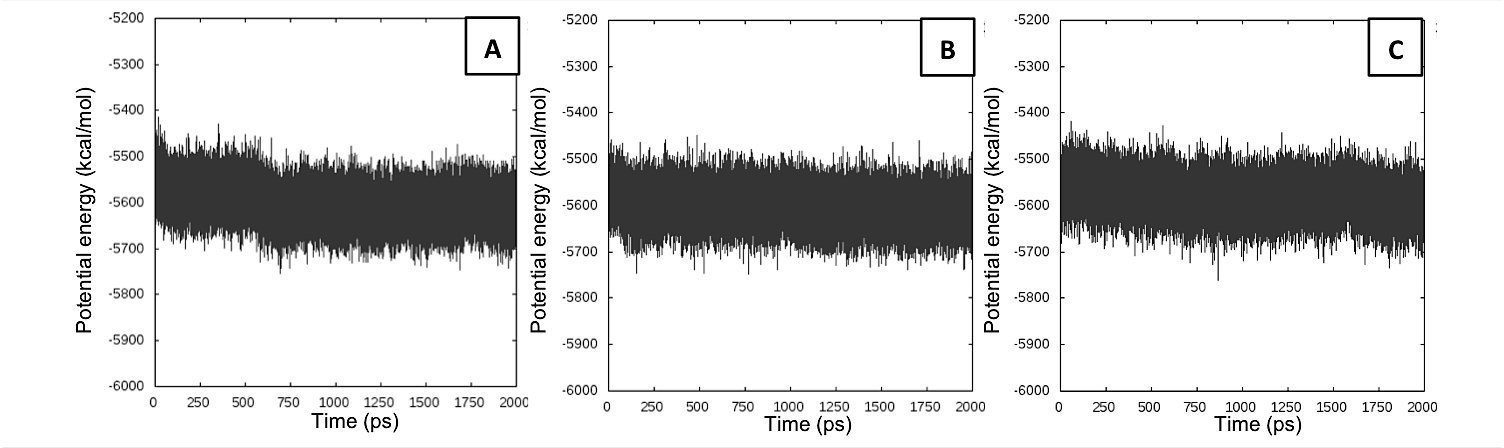

Supplement: Figure S3 — Potential energies (kcal/mol) for SULT1A3 isoform. A: The potential energy of the MD run 1. B: The potential energy of the MD run 2. C: The potential energy of the MD run 3. (TIFF) [file pone.0073587.s006.tiff]

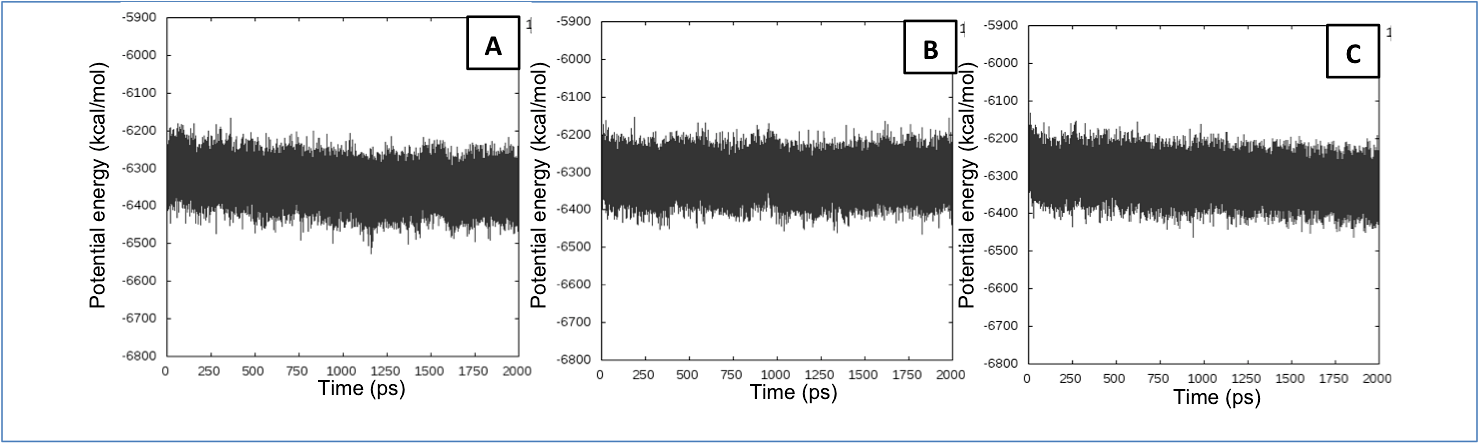

Supplement: Figure S4 — Potential energies (kcal/mol) for SULT1E1 isoform. A: The potential energy of the MD run 1. B: The potential energy of the MD run 2. C: The potential energy of the MD run 3. (TIFF) [file pone.0073587.s007.tiff]
