# Supplementary material for: In Silico Mechanistic Profiling to Probe Small Molecule Binding to Sulfotransferases
Source: PLoS One. 2013 Sep 6;8(9):e73587. doi: 10.1371/journal.pone.0073587 (PMC3765257; doi:10.1371/journal.pone.0073587)
Supplement: Table S1 — Protein-ligand interaction energies for active centroids as predicted by docking, averaged for the X-ray and the two best performing MD structures, and compared to the experimental affinities. (DOCX) [file pone.0073587.s008.docx]

| Isoform | Compound ID | Protein-ligand interaction energies (kcal/mol) | IC50 (M) | Km (M) |
| --- | --- | --- | --- | --- |
| SULT1A1 | 681 | -5.9 | NA | 130e-6 |
|  | 1864 | -7.2 | NA | 18e-6 |
|  | 3371 | -9.0 | 1.5 e-6 | NA |
|  | 3672 | -7.3 | 159e-6 | NA |
|  | 3715 | -5.5 | 74e-6 | NA |
|  | 3825 | -9.3 | 41e-6 | NA |
|  | 3826 | -7.2 | 245e-6 | NA |
|  | 4044 | -7.7 | 20e-9 | NA |
|  | 4495 | -7.6 | 5.4e-6 | NA |
|  | 5564 | -6.9 | 2.3e-6 | NA |
|  | 5591 | -6.8 | NA | 5.4e-6 |
|  | 5699 | -7.4 | NA | 43.3e-6 |
|  | 6623 | -7.3 | NA | 4.2e-6 |
|  | 11349 | -7.7 | 1.6e-6 | NA |
|  | 72276 | -6.5 | 6.1e-6 | 10e-6 |
|  | 119346 | -6.2 | 1.9e-6 | NA |
|  | 242834 | -6.9 | NA | 141e-9 |
|  | 3015395 | -6.9 | 400e-9 | NA |
|  | 5281607 | -7.4 | NA | 2.5e-6 |
|  | 5281708 | -7.5 | NA | 1.7e-6 |
| SULT1A3 | 1864 | -7.1 | NA | 65e-6 |
|  | 3059 | -7.8 | 79e-6 | NA |
|  | 3715 | -8.4 | 103e-6 | NA |
|  | 4044 | -8.2 | 150e-9 | NA |
|  | 4488 | -8.2 | 180e-6 | NA |
|  | 5991 | -10.3 | NA | 18.9e-9 |
|  | 9064 | -8.1 | NA | 87e-6 |
|  | 12066 | -7.9 | 142e-6 | NA |
|  | 445154 | -7.6 | NA | 1.3e-6 |
|  | 969516 | -7.4 | 4e-6 | NA |
| SULT1E1 | 5280443 | -5.6 | NA | 5.3e-6 |
|  | 5281607 | -8.6 | NA | 4e-6 |
|  | 5281708 | -8.7 | NA | 3e-6 |
|  | 20541757 | -9.8 | NA | 700e-9 |

**Table S1.** Protein-ligand interaction energies for active centroids as predicted by docking, averaged for the X-ray and the two best performing MD structures, and compared to the experimental affinities.
