# Supplementary material for: In Silico Mechanistic Profiling to Probe Small Molecule Binding to Sulfotransferases
Source: PLoS One. 2013 Sep 6;8(9):e73587. doi: 10.1371/journal.pone.0073587 (PMC3765257; doi:10.1371/journal.pone.0073587)
Supplement: Table S2 — Performance of QSAR models built by using support vector machine, random forest, and naïve Bayes machine-learning methods. (DOCX) [file pone.0073587.s009.docx]

**Table S1.** Performance of QSAR models built by using support vector machine (SVM), random forest, and naïve Bayes machine-learning methods.

| Isoform | Accuracy without binding energy  SVM | Accuracy including binding energy  SVM | Accuracy without binding energy  Random Forest | Accuracy including binding energy  Random Forest | Accuracy without binding energy  Naïve Bayes | Accuracy including binding energy  Naïve Bayes |
| --- | --- | --- | --- | --- | --- | --- |
| SULT1E1 | 73.94% | 75.46% | 64.24% | 72.12% | 72.12% | 72.12% |
| SULT1A3 | 73.80% | 78.00% | 70.40% | 77.40% | 72.40% | 74.40% |
| SULT1A1 | 60.85% | 67.28% | 54.75% | 59.32% | 65.73% | 66.44% |
