# Supplementary material for: In Silico Mechanistic Profiling to Probe Small Molecule Binding to Sulfotransferases
Source: PLoS One. 2013 Sep 6;8(9):e73587. doi: 10.1371/journal.pone.0073587 (PMC3765257; doi:10.1371/journal.pone.0073587)
Supplement: Text S1 — Drug-like filter parameters and binding sites’ residues. (DOCX) [file pone.0073587.s010.docx]

**Drug-like filter.**

Parameters used: molecular weight between 150 and 800 Da, number of hydrogen bond donors between 0 and 6, number of hydrogen bond acceptors between 0 and 10, number of rotatable bond between 0 and 15, number of rings between 0 and 7, size of the largest ring 18, polar surface are between 0 and 160, logP between -4 and 6, number of formal charges between 0 and 4, sum of formal charges between -3 and 3.

**Binding pocket residues.**

SULT1A1: I21, F24, P47, K48, T51, T52, F76, M77, F81, F84, I89, P90, G92, T95, K106, H108, S138, H141, M145, K147, V148, V167, S168, N239, T242, V243, E246, F247, P254

SULT1A3: I21, Y23, F24, P47, K48, G50, T51, T52, S55, I75, Y76, V77, V79, P80, F81, V84, D86, E89, P90, S91, G92, T95, K106, H108, Y139, F142, E146, A148, H149, S168, Y169, M237, Y240, T242, V243, P244, L247, M248, F255

SULT1E1: Y20, F23, P46, K47, G49, T50, T51, S54, I74, F75, I78, F80, C83, K105, H107, F138, F141, V145, A146, G147, H148, Y168, Y239, L242, I246, M247, F254
